# Comparison of the Phenolic Content and Antioxidant Activities of *Apocynum venetum* L. (Luo-Bu-Ma) and Two of Its Alternative Species

**DOI:** 10.3390/ijms11114452

**Published:** 2010-11-09

**Authors:** Taigang Liang, Wenyan Yue, Qingshan Li

**Affiliations:** School of Pharmaceutical Science, Shanxi Medical University, No 56, Xinjian Nan Road, Taiyuan 030001, Shanxi, China; E-Mails: ltaigang@gmail.com (T.L.); yuewenyanhappy@163.com (W.Y.)

**Keywords:** *Apocynum venetum* L., *Poacynum pictum* (Schrenk) Baill., *Poacynum hendersonii* (Hook.f.) Woodson, antioxidant activity, phenolic, flavonoid, HPLC

## Abstract

The leaves of *Apocynum venetum* L. (AV), a native Chinese plant, have been used as folk medicine in China and Japan. This study evaluated the content of the active antioxidant component and antioxidant activities of AV, and its two alternative species, *Poacynum pictum* (Schrenk) Baill. (PP) and *Poacynum hendersonii* (Hook.f.) Woodson (PH). The total phenolic and total flavonoid contents were determined. In addition, the quantitative analysis of two major flavonoid compounds (hyperoside and isoquercitrin) was carried out by HPLC. The antioxidant activities were investigated by the 1,1-diphenyl-2-picrylhydrazyl (DPPH) radical scavenging activity method, the reducing power test and the chelating ability of ferrous ions. The highest total phenolic and flavonoid contents were observed in the AV methanolic extract, followed by the PP and PH methanolic extracts. HPLC analysis indicated that isoquercitrin was one of the major components in all three species, however, hyperoside was only detected in AV at high levels. All the antioxidant assays we performed demonstrated that the AV extract was markedly superior to those of the other two species.

## 1. Introduction

Many investigations have demonstrated that the overproduction of reactive oxygen species (ROS), which includes the production of hydroxyl radicals, superoxide anions, and hydrogen peroxide, can contribute to DNA damage, protein oxidation and lipid peroxidation in living tissues and cells [1–3]. ROS overproduction has been linked to many clinical diseases, such as heart disease, diabetes, liver injury, cancer and aging [4–8]. The innate defense may not be sufficient to combat severe or continued oxidative stress. Hence, the input of exogenous antioxidants is constantly required to maintain an adequate level of antioxidants to balance the ROS in the human body [9].

Nowadays, it is generally agreed that synthetic antioxidants such as butylhydroxyanisole (BHA) and butylhydroxytoluene (BHT) need to be replaced with natural antioxidants because of their potential health risks and toxicity [10,11]. Thus, the use of naturally occurring antioxidants, mainly phenolic compounds, has attracted considerable attention given their comparative safety. The antioxidant capacity of phenolic compounds is mainly due to their redox properties, allowing them to act as reducing agents, hydrogen donors, singlet oxygen quenchers or metal chelators.

As an important source of natural antioxidants, many traditional Chinese medicinal herbs have proven to exhibit strong antioxidant activity [12,13]. *Apocynum venetum* L. (Luobuma in Chinese, AV) is a wild shrub widely distributed in middle and northwestern China. Its leaves have been used as tea in China and Japan for hundreds of years. In traditional Chinese medicine, AV is generally used to treat hypertension, nephrosis, neurasthenia and hepatitis [14]. Recently, extracts of the AV leaf were assigned multiple biological functions, such as antidepressant [15], anti-anxiety and antihypertensive activities [16]. Additionally, enhanced abilities for peroxynitrite-scavenging and the protection of lipid hydroperoxide-induced species in PC-12 cells were also observed [17,18]. Many phenolic compounds, mainly flavonoids, have been isolated from the leaves of AV; among them, hyperoside and isoquercitrin were the major flavonoid compounds [19]. New reports have demonstrated that both hyperoside and isoquercitrin exhibit potent antioxidant activities [20,21].

In addition to AV, the leaves of *Poacynum pictum* (Schrenk) Baill. (PP) and *Poacynum hendersonii* (Hook.f.) Woodson. (PH) are also available in some local markets as substitutes of AV.

To systematically evaluate the antioxidant properties of the three species, the total phenolic and total flavonoid contents were determined; the quantitative analysis of the two potent antioxidant flavonoids, namely hyperoside and isoquercitrin, was also carried out by HPLC. The antioxidant activities were investigated by the 1,1-diphenyl-2-picrylhydrazyl (DPPH) radical scavenging activity method, the reducing power test, and the chelating ability of ferrous ions.

## 2. Results and Discussion

### 2.1. Total Phenolic and Total Flavonoid Content

The content of extractable phenolic compounds in methanolic extracts was determined by the Folin-Ciocalteau method. The results, given in Table 1, show that the total phenolic content of AV (92.71 ± 2.16 mg/g) was markedly higher than that of PP and PH (53.58 ± 0.81 mg/g and 50.61 ± 1.19 mg/g), expressed as mg gallic acid equivalents (GAE) per g dry weight.

Flavonoids are the most common and widely distributed group of plant phenolic compounds and are usually very effective antioxidants [28,29]. In this study, the total flavonoid content of methanolic extracts from leaves of three plant species were evaluated by the aluminum colorimetric assay. Rutin was used as a standard, and the total flavonoid content of the extracts was expressed in milligram of rutin equivalents per gram of dry extract (mg RE/g dry extract). The total flavonoid content of the three extracts varied considerably and ranged from 17.02 to 31.09 mg RE/g dry extract. The data presented in Table 1 indicate that the highest content of total flavonoid was present in extracts obtained from AV (31.09 ± 1.73 mg RE/g), followed by PH (18.16 ± 1.04 mg RE/g) and PP (17.02 ± 1.35 mg RE/g).

This result shows that both total phenolic and total flavonoid contents in AV were significantly higher (*P* < 0.05) than those in PP and PH. On the other hand, flavonoids represent the main group of phenolic compounds in all three plants. Previous phytochemistry investigations have demonstrated that AV possesses many phenolic and flavonoid compounds [30–32]. In the preliminary study, 70% methanol was found to be more effective in recovering amounts of phenolic and flavonoid compounds from three plants than other solvents. So, 70% methanol was used as extraction solvent to prepare the extracts.

### 2.2. Quantitative Analysis of Hyperoside and Isoquercitrin

**Figure 1.**
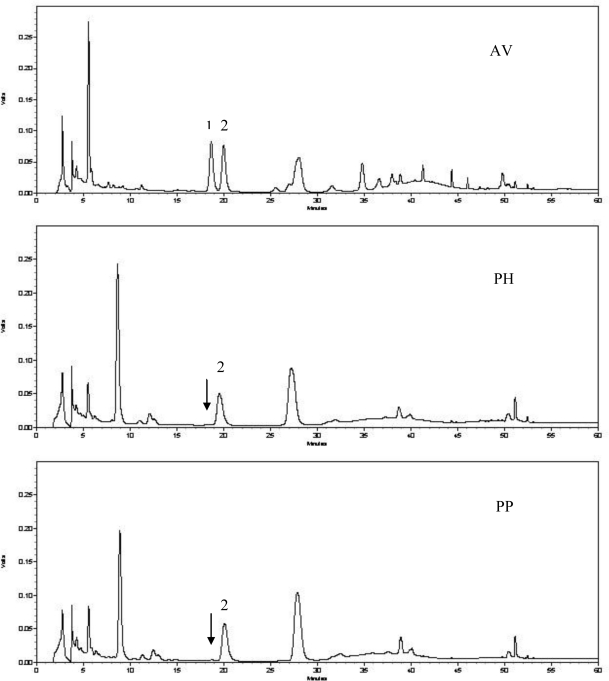
Typical HPLC chromatograms in the leaves of three species with detector responses at 360 nm (1: hyperoside, 2: isoquercitrin).

In the present study, the HPLC data from the quantitative analyses of hyperoside and isoquercitrin from extracts of three plant species are presented in Table 2. The chromatograms with the detector responses at 360 nm are shown in Figure 1. Two flavonoid compounds namely, hyperoside and isoquercitrin, were identified by comparison to the retention times of the authentic standards analyzed under identical analytical conditions. As seen in Figure 1, the chromatogram of AV exhibited some differences compared to the other two species, while PP and PH were very similar in their chromatographic profiles. The results of this quantitative analysis demonstrated that isoquercitrin was the predominant flavonoid compound in all three species; its contents were 13.62 ± 1.24 mg/g dry extract of AV, 11.89 ± 1.13 mg/g dry extract of PP and 12.10 ± 0.81 mg/g dry extract of PH, respectively (Table 2).

However, hyperoside was only present in the AV extract at the high concentration of 11.61 ± 1.02 mg/g dry extract and was not detected in PP and PH.

It has been confirmed that flavonoids as active constituents were responsible for antioxidant activity [33,34]. Apart from hyperoside and isoquercitrin, some other flavonoids such as rutin and quercetin have been determined in AV by HPLC [35]. In this study, HPLC analysis indicated that there were significant differences between AV and the other two species, especially with regard to hyperoside and isoquercitrin contents. It can be concluded, therefore, that their antioxidant activities apparently varied.

### 2.3. DPPH Radical Scavenging Activities

**Figure 2.**
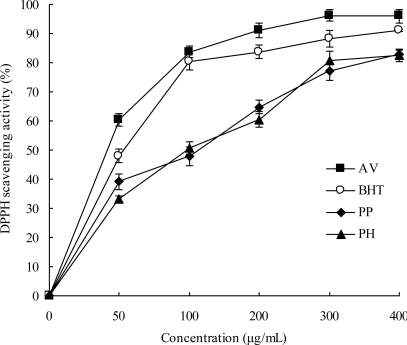
DPPH free radical scavenging activity in leaves of three species. Results are mean ± SD (*n* = 3).

The free radical-scavenging activities of the leaf methanol extracts of three species, along with the reference standard BHT, were determined by the DPPH method; the results are given in Figure 2. AV was the most active DPPH radical scavenger (IC_50_, 33.72 μg/mL), and was superior to the positive control, BHT (IC_50_, 43.16 μg/mL); it was followed by PP (IC_50_, 92.01 μg/mL) and PH (IC_50_, 99.83 μg/mL). Each extract exhibited a dose-response relationship of DPPH radical-scavenging activity. At a concentration of 200 μg/mL, AV possessed a DPPH radical-scavenging activity above 90%; however, PP and PH only showed an inhibition rate of about 60%. The strong DPPH scavenging activity of AV could be attributed to the higher phenolic and flavonoid content, especially since it contained the potent radical scavengers hyperoside in abundant amounts (IC_50_, 1.31 μg/mL) [20].

The DPPH method has been widely used to test the free radical-scavenging ability of various samples [36]. In this study, AV exhibited high DPPH free radical-scavenging capacity (IC_50_, 33.72 μg/mL). Although its value was lower than that reported for green tea methanolic extract (IC_50_, 12.70 μg/mL) [37] which has been a well-known antioxidant, AV contains no caffeine and theophylline and can not induce adverse effects such as sleeplessness, tachycardia, *etc.* [38]. In addition, Yokozawa *et al.* [17] documented that AV possesses peroxynitrite-scavenging activity. Thus, these findings suggested that AV has good free radical scavenging ability and can be used as a radical inhibitor or scavenger.

### 2.4. Reducing Power

Reducing power is widely used to evaluate the antioxidant activity of polyphenols. The reducing power is generally associated with the presence of reductones, which exert antioxidant action by breaking the free radical chain and by donating a hydrogen atom [39].

**Figure 3.**
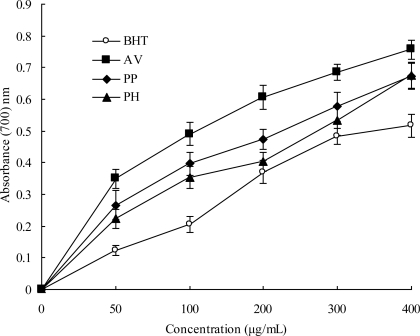
Reducing power in leaves of three species. Results are mean ± SD (*n* = 3).

As shown in Figure 3, extracts of AV showed the highest reducing power, while the PH and PP extracts exhibited activities of similar reducing power.

At a concentration of 50–400 μg/mL, the reducing power of extracts followed the order of AV > PP ≥ PH; extracts of all three species exhibited higher reducing power than BHT, suggesting that they possess a stronger electron-donating capacity.

In the early papers, a direct correlation between reducing capacity and antioxidant activities of certain plant extracts was reported [40,41]. Here, the data on the reducing power of three extracts suggest that it is likely to contribute significantly towards the antioxidant effect. Some foods or medicinal plants for example Chinese water chestnut and *rhubarb*, were observed to exhibit stronger reducing activity than BHT [42,43]. As naturally occurring antioxidants, they have received growing interest.

### 2.5. Chelating Ability of Ferrous Ions

**Figure 4.**
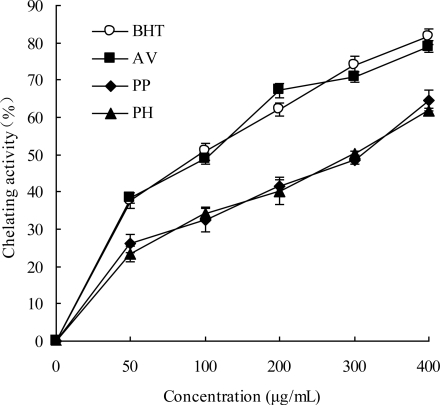
Chelating ability in leaves of three species. Results are mean ± SD (*n* = 3).

Transition metals have been proposed to be the catalysts for the initial formation of radicals. Chelating agents may stabilize transition metals in living systems and inhibit the generation of radicals, thereby reducing free radical damage [44]. Thus, the chelating ability of transition metals is an important mechanism of antioxidant activity. As seen in Figure 4, all the extracts demonstrated the ability to chelate iron ions. Among the three extracts, AV displayed a similar ferrous ion chelating activity (38.47–79.01%) to BHT (37.70–81.59%) at concentrations of 50–400 μg/mL and was followed by PP (26.22–64.51%) and PH (23.47–61.71%). In this assay, the chelating activities of all extracts increased in a dose-dependent manner and were associated with the total phenolic and flavonoid contents.

AV contained plenty of phenolic and flavonoid compounds which may be responsible for the chelating ability of ferrous ions due to the presence of a hydroxyl group and electron-donating methoxy group in their structures [45]. Futhrermore, Hudson and Lewis [46] reported that a carbonyl at the 4 position as well as 3- or 5-hydroxyl groups in flavonoids were important for the metal chelating activity. The two major flavonoids in AV, hyperoside and isoquercitrin, both contain the 4-carbonyl and 5-hydroxyl groups, which could explain to some extent why AV possessed relatively higher activity.

## 3. Experimental Section

### 3.1. Plant Materials

The leaves of *Apocynum venetum* L. and *Poacynum pictum* (Schrenk) Baill. were collected from Shanxi Province, China, in July 2006. The leaves of *Poacynum hendersonii* (Hook.f.) Woodson were collected from Qinhai Provence, China, in July 2007. The voucher specimens were identified by Dr. Jianping Gao (School of Pharmaceutical Science, Shanxi Medical University, China)

### 3.2. Standards and Reagents

Folin-Ciocalteu reagent, 1,1-diphenyl-2-picrylhydrazyl (DPPH), Butylated hydroxytoluene (BHT), and isoquercitrin were purchased from Sigma Chemicals Co. (St. Louis, MO, U.S.), and hyperoside, gallic acid, rutin, obtained from the National Institute for the Control of Pharmaceutical and Biological Products (Beijing, China), purity was >98%. Other chemicals in the studies were of highest quality commercially available from local suppliers (Shanghai, China).

### 3.3. Equipment and Apparatus

High-performance liquid chromatography system (Shimadzu Corporation, Kyoto, Japan); UV-1100 spectrophotometer (Beijing Rayleigh Analytical Instrument Corporation, Beijing, China); RE-52A rotary evaporators (Shanghai Yarong Biochemistry Instrument Factory, Shanghai, China).

### 3.4. Preparation of Methanolic Extracts

Dried powders of ground leaves (5 g) were extracted by ultrasonic bath with 50 mL of 70% methanol at room temperature for 30 min by the method of Wang *et al.* [22]. The extract was filtered through Whatman No.4 filter paper. The residues were re-extracted in the same manner. After that, the two filtrates were combined and evaporated under reduced pressure using a rotary vacuum-evaporator at 50 °C. The resulting dry materials were collected and stored at 4 °C in the dark for further analysis. Light exposure was avoided throughout the extraction process.

### 3.5. Determination of Total Phenolics Content

Total phenolic contents were assayed using Folin-Ciocalteu reagent according to the method of Slinkard and Singleton [23] with some modifications. Briefly, 0.1 mL of sample, 1.9 mL distilled water and 1.0 mL of Folin-Ciocalteau reagent were seeded in a tube, and then 1.0 mL of 20% Na_2_CO_3_ was added. The reaction mixture was incubated at 25 °C for 2 h and the absorbance of the sample was recorded at 765 nm against the reagent blank. The sample was tested in triplicate and a calibration curve for gallic acid was obtained. The results were compared to gallic acid calibration curve and the total phenolic content of extract was expressed as mg of gallic acid equivalents (GAE) per gram of dry extract.

### 3.6. Determination of Total Flavonoids Content

The spectrophotometer assay for the quantitative determination of flavonoid content was carried out as described by Wang *et al*. [24] with minor modifications using rutin as a standard. Briefly, extracts or standard solutions (0.25 mL) were mixed with 1.25 mL distilled water and 75 μL 5% NaNO_2_. After 6 min, 75 μL of 10% AlCl_3_ was added. After another 5 min, 0.5 mL of 1 M NaOH was added to the mixture. Immediately, the absorbance of the mixture was determined at 510 nm *versus* prepared water blank. Total flavonoids content was expressed as mg rutin equivalents (RE) per gram dry extract.

### 3.7. HPLC Determination of Hyperoside and Isoquercitrin

Quantitative HPLC analysis was performed on a high-performance liquid chromatography system equipped with two LC-10 AT*VP* pumps, a variable UV-VIS detector (SPD-10A*VP*) and CLASS-*VP* software for data acquisition and processing. A reversed-phase Kromasil C_18_ column (150 × 4.6 mm, particle size 5 μm) was used for separation. The mobile phase was composed of solvent A (acetonitrile containing 0.05% phosphoric acid), and solvent B (water containing 0.05% phosphoric acid). The linear gradient was from 17% to 100% of solvent A in 45 min; this proportion was held up to 60 min, while the flow rate was set at 1.0 mL/min; the column temperature was maintained at 25 °C, and UV detection was performed at 360 nm with a 20 μL injection volume. The standard solutions of hyperoside and isoquercitrin were prepared as serial dilutions to obtain a calibration curve. The extracts were dissolved in methanol-water (50:50) and were filtered through a nylon membrane filter (0.45 μm) before injection. Hyperoside and isoquercitrin were identified by their retention times after comparison with authentic markers and quantified by an external assay.

### 3.8. DPPH Radical Scavenging Activity

Free radical scavenging activity of the three species extracts and BHT were determined using a stable 1,1-diphenyl-2-picrylhydrazyl radical (DPPH) [25]. DPPH is a free radical of violet color. The antioxidants in the sample scavenge the free radicals and turn it into yellow colour. The change of color from violet to yellow is proportional to the radical scavenging activity.

Briefly, 1 mM solution of DPPH in ethanol was prepared, and 4 mL of this solution was mixed with 1 mL of extract solution at various concentrations immediately and then incubated for 30 min at room temperature. The absorbance of the sample was measured at 517 nm. Radical scavenging activity was expressed as the inhibition percentage (IP) of free radical and was calculated using the formula:

$$\text{IP}(\%)\;=\;([{\text{A}}_{\text{control}}\;-{\text{ A}}_{\text{test}})/{\text{A}}_{\text{control}}])\;\times \;100\%$$

Where A_control_ is the absorbance of the control reaction (containing all reagents except the tested extracts), and A_test_ is the absorbance of the test extract. Scavenging activity of the plant extracts was also estimated based on the percentage of the DPPH reduction by calculating the IC_50_ values (concentration in μg/mL that caused 50% inhibition of DPPH radicals) using a non-linear regression analysis.

### 3.9. Reducing Power

The reducing powers of the extracts and BHT were determined according to the method described by Zhu *et al.* [26]. A 0.1 mL aliquot of each extract (50–400 μg/mL) and BHT were mixed with an equal volume of 0.2 M phosphate buffer (pH 6.6) and 1% potassium ferricyanide [K_3_Fe(CN)_6_], and then incubated at 50 °C for 20 min. A portion (2.5 mL) of 1% trichloroacetic acid was added to the mixture to stop the reaction, and then the mixture was centrifuged at 1000 rpm for 10 min. The supernatant (0.25 mL) was mixed with 0.25 mL distilled water and 0.1% FeCl_3_ (0.5 mL) and then the absorbance was measured at 700 nm. The reducing powers of the tested samples increased with the absorbance values.

### 3.10. Chelating Ability of Ferrous Ions

Chelating ability was determined by the method of Dinis *et al*. [27] with slight modification. The Fe^2+^ level was monitored by measuring the formation of the ferrous ion-ferrozine complex. The methanol extract (1.0 mL) was mixed with methanol (3.7 mL), 2 mM FeCl_2_ (0.1 mL) and 5 mM ferrozine (0.2 mL) and the mixture was shaken and left at room temperature for 10 min. The absorbance of the resulting solution was measured at 562 nm against the blank performed in the same way using FeCl_2_ and water. The percentage of inhibition of ferrozine-Fe^2+^ complex formation was calculated using the equation:

$$\text{Chelating ability }(\%)\;=\;([{\text{A}}_{\text{control}}\;-{\text{ A}}_{\text{test}})/{\text{A}}_{\text{control}}])\;\times \;100\%$$

Where A_control_ is the absorbance of the control reaction (containing all reagents except the tested extracts), and A_test_ is the absorbance of the test extract or BHT. All tests were performed in triplicate.

### 3.11. Statistical Analysis

All the experimental results were centered using three parallel measurements of the mean ± SD. Analysis of variance (ANOVA) was performed. Duncan’s new multiple-range test was used to determine the differences in means. All *P* values less than 0.05 were regarded as significant.

## 4. Conclusions

In the present study, the AV extract demonstrated the highest content of total phenolics and total flavonoids; it also contained the two abundant flavonoids, hyperoside and isoquercitrin, but hyperoside was not detected in the extracts of PP and PH. The three antioxidant assays, namely the DPPH radical scavenging activity method, the reducing power test and the chelating ability of ferrous ions, showed that AV was markedly superior to the two alternative species. The high antioxidant activity of AV was attributed to its higher total phenolic and flavonoid contents. Further, it can be speculated that this excellent effect may be related to a high content of strong radical scavenger-hyperoside that only existed in the AV methanolic extract.

## Figures and Tables

**Table 1.** Total phenolics and flavonoids contents of leaves of three species (*n* = 3). Values are the mean ± SD of three parallel measurements; different letters in the same column indicate significant differences (*P* < 0.05).

**Plant**	**Total phenolics content** (**mg GAE/g dry extract**)	**Total flavonoids content** (**mg RE/g dry extract**)
**AV**	92.71 ± 2.16^a^	31.09 ± 1.73^a^
**PP**	53.58 ± 0.81^b^	17.02 ± 1.35^b^
**PH**	50.61 ± 1.19^b^	18.16 ± 1.04^b^

*GAE*: gallic acid equivalents, *RE*: rutin equivalents.

**Table 2.** The hyperoside and isoquercitrin contents of leaves of three species (*n* = 3). ND: not detected. Values are the mean ± SD of three parallel measurements; different letters in the same column indicate significant differences (*P* < 0.05).

**Plant**	**Hyperoside** (**mg/g dry extract**)	**Isoquercitrin** (**mg/g dry extract**)
**AV**	11.61 ± 1.02	13.62 ± 1.24^a^
**PP**	ND	11.89 ± 1.13^b^
**PH**	ND	12.10 ± 0.81^b^
